# Combating Age-Associated Immune Decline Using Kynurenine Pathway Interventions

**DOI:** 10.1093/geroni/igab046.2541

**Published:** 2021-12-17

**Authors:** Luis Espejo, Destiny DeNicola, Sam Freitas, Hope Dang, Emily Turner, Raul Castro-Portuguez, Anne Haskins, George Sutphin

**Affiliations:** 1 UNIVERSITY OF ARIZONA, TUCSON, Arizona, United States; 2 The University of Arizona, Tucson, Arizona, United States; 3 University of Arizona, Tucson, Arizona, United States

## Abstract

Select kynurenine pathway interventions extend lifespan in invertebrate models and are of interest in treating age-associated diseases. Kynurenine pathway activity is responsive to inflammatory signaling, and we are evaluating the potential for these interventions to increase pathogen resistance and curtail age-associated immune decline in Caenorhabditis elegans and mammals. The kynurenine pathway facilitates the catabolism of tryptophan to nicotinamide adenine dinucleotide (NAD). Our lab has found that supplementing the kynurenine metabolite 3-hydroxyanthranilic acid (3HAA) or inhibiting the enzyme 3HAA dioxygenase (HAAO) extends lifespan in C. elegans. 3HAA has demonstrated pro/anti-inflammatory properties in mammals, suggesting a potential role in immune function. C. elegans have a primitive immune system that lacks an adaptive element, but it recapitulates aspects of innate immune signaling and pathogen response. I hypothesize kynurenine pathway interventions that impact C. elegans’ lifespan similarly improve pathogen resistance and immunity. Interventions within the kynurenine pathway are capable of differentially impacting pathogenesis and lifespan of C. elegans challenged with Psuedomonas aeruginosa. C. elegans subjected to select lifespan-extending kynurenine pathway interventions fared better when challenged with P. aeruginosa at older ages. Additionally, fluorescent infection tracking has displayed decreased infection rates in worms with elevated 3HAA. Our data suggests pro-immune activity is facilitated by 3HAA acting downstream of the dbl-1 pathway in addition to directly inhibiting bacterial growth. Our goal is to discover the mechanism(s) through which the kynurenine pathway interacts with immune function in animals and identify potential targets for clinical therapy in aging populations.

